# A visualized bibliometric analysis of *Salvia miltiorrhiza* (Danshen) research: Trends, hotspots, and emerging frontiers

**DOI:** 10.1097/MD.0000000000046976

**Published:** 2026-01-30

**Authors:** Huan-Huan Li, Fei-Fei Bu, Chuan-Yao Zhang, Wen-Li Zhang, Peng Wang

**Affiliations:** a College of Traditional Chinese Medicine, Anhui University of Chinese Medicine, Hefei, China.

**Keywords:** bibliometric analysis, biosynthesis, clinical translation, pharmacology, *Salvia miltiorrhiza* (Danshen)

## Abstract

**Background:**

*Salvia miltiorrhiza* (Danshen) has shown significant therapeutic potential in cardiovascular and neurological disorders, highlighting its clinical importance. With the growing global attention on Danshen, a comprehensive bibliometric evaluation is needed to clarify the current research landscape and promote its international development. This study analyzed collaboration networks, research status, and emerging trends in Danshen-related studies.

**Methods:**

Publications on Danshen from 2006 to 2025 were retrieved from the Web of Science Core Collection. Original research articles were analyzed using CiteSpace, VOSviewer, and Bibliometrix to evaluate coauthorship networks, journal distribution, keyword co-occurrence, citation bursts, and co-citation patterns, thereby identifying leading contributors, emerging hotspots, and the intellectual foundations of the field.

**Results:**

Four thousand one hundred sixty-nine articles were retrieved, steadily increasing from 2006 to 2024. China dominated output (3706 publications), followed by the United States and South Korea, with China–United States and China–South Korea collaborations being most prominent. The Chinese Academy of Sciences was the most productive institution, and Liang Zongsuo was the leading individual contributor (70 papers). The *Journal of Ethnopharmacology* served as the leading publication platform. Research hotspots focused on the biosynthesis of tanshinones and phenolic acids and molecular mechanisms such as AKT and Nrf2 signaling, with emerging trends in network pharmacology and molecular docking.

**Conclusion:**

This study provides a comprehensive knowledge map of Danshen research, illustrating global collaboration, research development, and emerging directions. While offering a reference for future studies, the findings also highlight the lack of high-quality clinical evidence, indicating the need for translational and multicenter clinical research.

## 1. Introduction

*Salvia miltiorrhiza* (Danshen) has been used for centuries in traditional medicine, particularly for cardiovascular and cerebrovascular disorders. It is officially listed in the Chinese Pharmacopoeia, broadly incorporated into clinical prescriptions across East Asia, and has gained growing recognition within international pharmacopeias and governance frameworks.^[1,2]^ Beyond crude decoctions, Danshen has been developed into a variety of clinical preparations, including Compound Danshen Dripping Pills,^[3]^ Suhexiang Pill,^[4]^ and CGplus,^[5]^ which are extensively applied in clinical practice for related vascular diseases. The large-scale clinical use of these formulations underscores the medical importance of Danshen and highlights its translational potential. Modern pharmacological research has identified multiple bioactive constituents from Danshen, particularly tanshinones and salvianolic acids, which exhibit antioxidant, anti-inflammatory, antithrombotic, vasodilatory, and endothelial-protective activities in diverse preclinical models.^[6-8]^ Such findings provide mechanistic explanations for Danshen’s traditional uses while broadening interest into new therapeutic areas such as fibrosis, neurodegeneration, cancer prevention, and metabolic regulation.^[9-11]^ Advances in extraction, purification, and analytical chemistry have further enabled more consistent characterization of Danshen-derived compounds, strengthening experimental reproducibility and supporting quality control standards.

Globalization has driven a rapid growth of Danshen-related research across diverse fields spanning pharmacology, systems biology, biotechnology, and materials science.^[12,13]^ The thematic focus has also shifted over time. Early investigations concentrated on cardiovascular function, whereas more recent work has expanded toward areas such as hepatic fibrosis, tumor biology, regenerative medicine, synthetic biology, and drug metabolism.^[14-17]^ Omics-based approaches, network pharmacology, and nanotechnology have also been increasingly applied to dissect Danshen’s complex bioactivities.^[18-20]^ Nevertheless, the literature remains fragmented across subfields, countries, and institutions. This fragmentation makes obtaining a coherent understanding of progress challenging and limits the ability to set clear research priorities. Therefore, a panoramic overview integrating collaboration networks, leading contributors, and thematic evolution is required to clarify how the knowledge structure has developed and where emerging fronts are forming.^[21]^

Bibliometric analysis offers powerful tools to address these challenges. Unlike traditional narrative reviews, which are often selective and prone to subjectivity, bibliometric approaches systematically evaluate large bodies of literature and provide quantitative insights into knowledge structures and developmental trajectories.^[22]^ Through coauthorship and co-citation networks, it is possible to identify influential countries, institutions, authors, and journals; keyword co-occurrence and burst detection highlight persistent hotspots and emerging topics; and trend analyses capture the rise or decline of specific themes. Such methods have been successfully applied in related fields, including other medicinal plants and natural products, demonstrating their value in uncovering research frontiers and guiding future directions.^[23,24]^ By combining complementary platforms such as CiteSpace, VOSviewer, and Bibliometrix, a robust and multidimensional knowledge map can be generated to support evidence-based decision-making.

In this context, the present study conducts a comprehensive bibliometric analysis of Danshen-related original research over the past 2 decades. The objectives are quantifying publication trends and citation patterns, identifying leading countries, institutions, authors, and journals, delineating research hotspots and emerging directions using keyword and co-citation analyses, and assessing the balance between mechanistic research and clinically oriented work. By outlining strengths and gaps in the current landscape, this study provides an evidence-based reference to guide future investigations, foster targeted collaboration, and support translational and multicenter clinical research design.

## 2. Materials and methods

### 2.1. Data collection

Web of Science is a widely recognized global platform covering natural sciences, social sciences, arts, and humanities, and provides a trusted citation database maintained by reputable publishers.^[25]^ We retrieved literature from the Web of Science Core Collection from January 2006 to February 2025 to ensure comprehensive and high-quality coverage. The search strategy employed the following terms: Topic = (“S*alvia miltiorrhiza*” OR “Danshen”). Data cleaning was conducted to maintain the focus and accuracy of the analysis: only original research articles were retained, while reviews, conference proceedings, book chapters, and retracted publications were excluded. After this screening, a total of 4169 valid records were identified. All records were exported with complete bibliographic information and cited references, saved in plain text format for subsequent bibliometric and visualization analysis.

As this study did not involve the examination or treatment of patients or review of patient records, it was exempt from review and approval by our research ethics committee, and informed consent was not applicable.

### 2.2. Data analysis

Bibliometric and network visualization analyses were performed using CiteSpace, VOSviewer, and bibliometrix. The dataset of 4169 original research articles was analyzed across multiple dimensions, including annual publication trends, country-level contributions, institutional affiliations, journal distribution, author productivity, and keyword co-occurrence. Visual network maps were generated for each dimension to provide an intuitive depiction of research patterns, collaborative networks, and thematic evolution. This approach enabled a systematic assessment of the research landscape, identifying key contributors, emerging hotspots, and trends in Danshen-related studies over the past 2 decades.

## 3. Results and analysis

### 3.1. Annual publication and citation statistics

**Figure 1.**
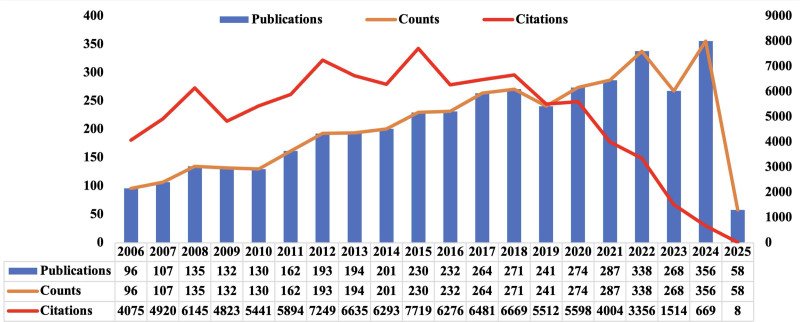
Publication trends and citation analysis of Danshen research (2006–2025).

4169 valid publications were included, contributed by 11,523 authors from 5957 institutions across 102 countries, and published in 917 journals (Table 1). Trends in publications and citations from 2006 to 2025 are shown in Figure 1. The annual number of publications exhibited a steady upward trajectory, peaking at 356 articles in 2024. In contrast, citation counts initially increased but later declined, reaching a maximum in 2015. Combined with Figure 5D, this decline may reflect shifts in research focus, greater interdisciplinary integration, and diversification of research topics. These trends suggest that future research should emphasize emerging areas, strengthen multidisciplinary collaboration, and integrate innovative methodologies to sustain progress and enhance the quality of Danshen research.

**Table 1:** The basic information of the included literature.

Category	Value	Category	Value
Time span	2006–2025	Average publication Period (yr)	8.08
Number of publications	4169	Number of sources	917
Number of authors	11,523	Annual growth rate	−2.62%
Proportion of international collaborative authorship	12.33%	Number of authors of single-authored documents	17
Number of author keywords	8523	Number of coauthors per document	7.14
Document average age	8.08	Number of references	1,13,017
Number of author keywords	8523	Average citations per document	23.81

**Figure 2.**
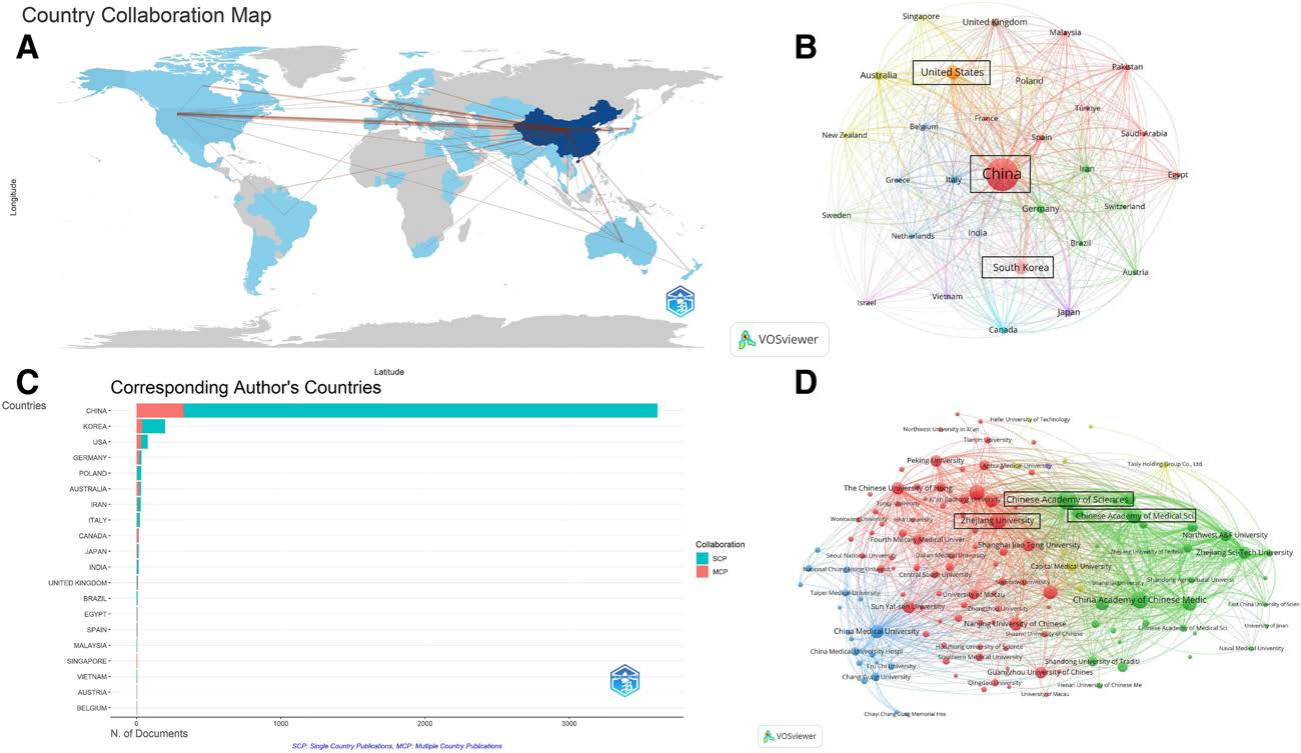
(A) Global collaboration map of international partnerships in Danshen research, generated with Bibliometrix. (B) Country-level collaboration network highlighting major contributing countries. (C) Distribution of publications by corresponding authors’ countries, created in Bibliometrix (red bars indicate multiple country publications, blue bars indicate single country publications). (D) An institutional collaboration network displays leading research institutions and their cooperative structures.

**Figure 3.**
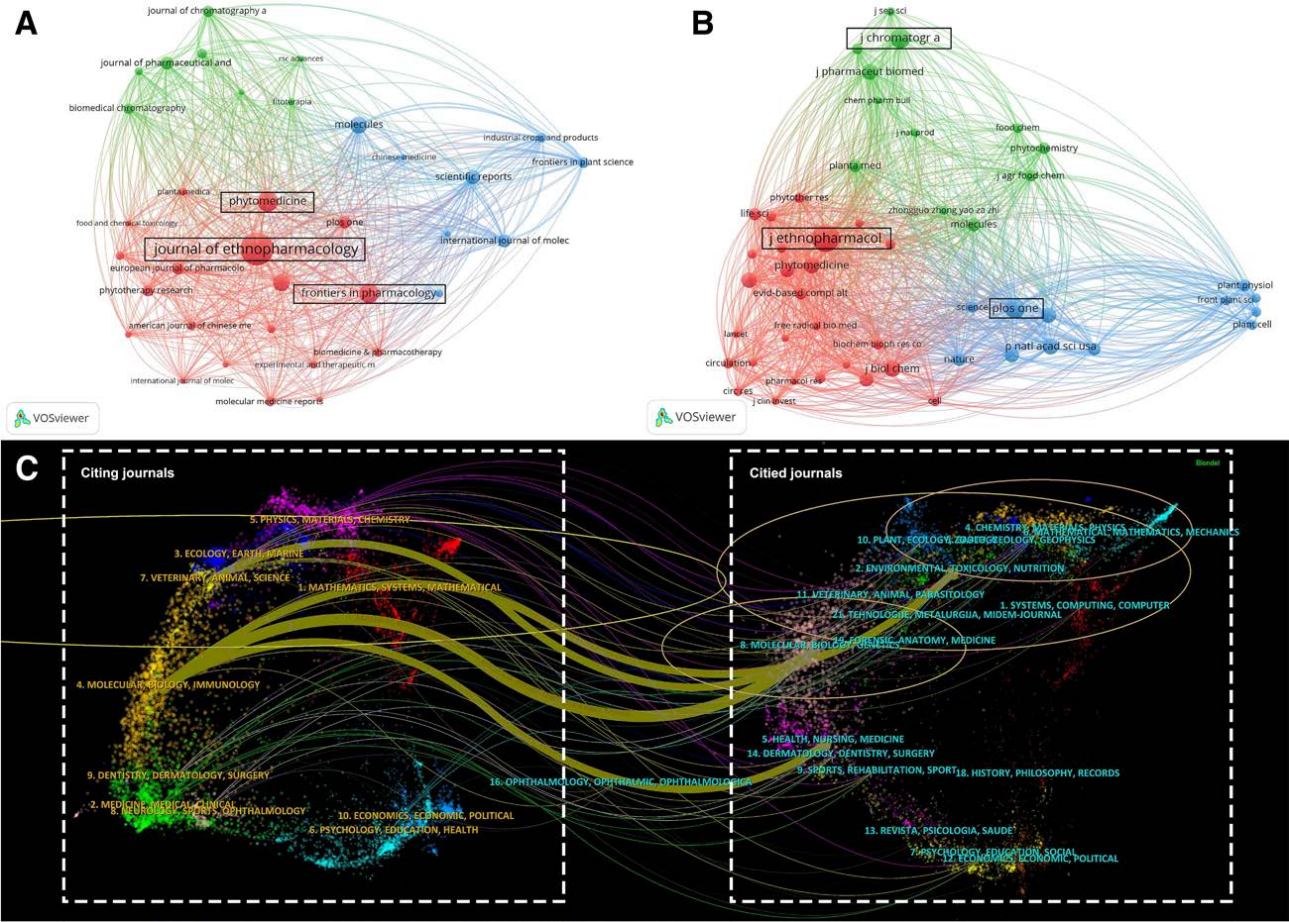
(A) Network map of journal collaboration in Danshen research. (B) Network map of journal co-citation relationships. (C) A dual-map overlay was generated with CiteSpace, illustrating interdisciplinary citation flows between citing and cited journals.

**Figure 4.**
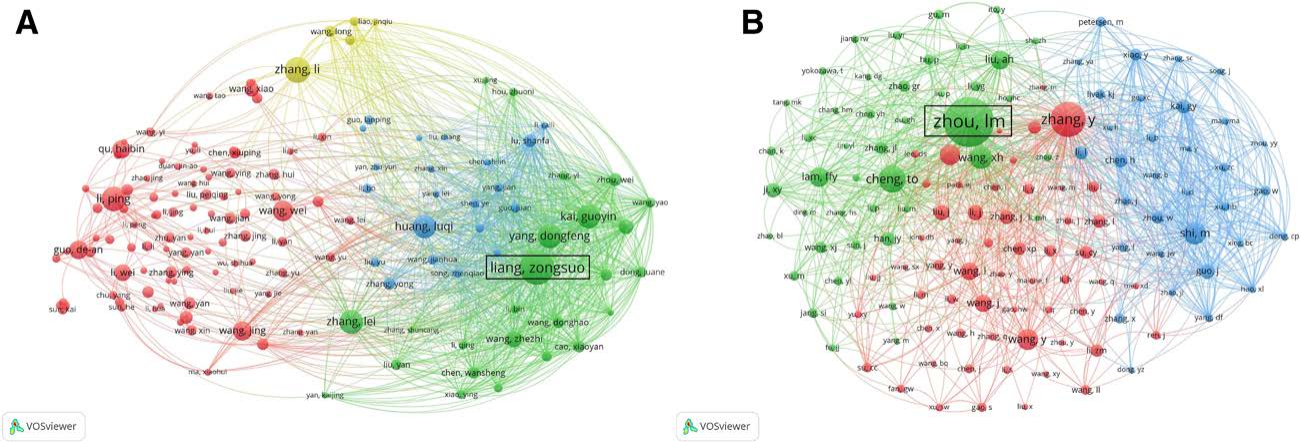
(A) Network map of author collaborations in Danshen research. (B) Network map of co-cited author relationships.

### 3.2. Analysis of countries and institutions

The global collaboration network in Danshen research reveals extensive international connections, with denser links reflecting stronger partnerships (Fig. 2A). China has developed broad collaborative relationships with multiple countries, among which the United States emerges as its most significant partner. The distribution of contributing countries highlights apparent disparities in output (Fig. 2B). China leads with 3706 publications, far exceeding those of other nations. With this dominance, China ranks first in single country publications and multiple country publications. An interesting observation is that while South Korea is behind the United States in total publications, it surpasses the United States in the number of documents by the corresponding author’s country (Fig. 2C). China’s influence has further facilitated the establishment of wide-ranging international partnerships. At the institutional level, several organizations stand out for their significant contributions (Fig. 2D). The Chinese Academy of Sciences, the China Academy of Chinese Medical Sciences, and Zhejiang University rank among the most productive institutions. These centers demonstrate a sustained commitment to advancing traditional Chinese medicine (TCM) research and actively foster academic exchange and collaborative progress, thereby enhancing the global visibility and impact of Danshen research.

### 3.3. Journal analysis

The distribution and influence of journals in Danshen research demonstrate distinct publication and citation patterns (Fig. 3). Each node represents a journal in the network visualization, and the connections indicate their degree of association. In terms of publication volume, the *Journal of Ethnopharmacology* ranks first with 222 articles, followed by *Phytomedicine* (115 articles) and *Frontiers in Pharmacology* (108 articles; Fig. 3A), highlighting the *Journal of Ethnopharmacology* as a central platform for disseminating research in this field. A similar trend is observed in the co-citation analysis, where the *Journal of Ethnopharmacology* recorded the highest citation frequency (3190 citations), followed by the *Journal of Chromatography A* and *PLoS One* (Fig. 3B). These findings emphasize these journals’ academic influence and recognition, indicating their pivotal role in shaping the knowledge base of Danshen research. The journal overlay visualization provides further insights into the disciplinary landscape. Journals publishing original research are positioned on the left, while co-cited journals appear on the right. The colors represent disciplinary categories, whereas node size and density reflect the volume of journals. Molecular Biology and Immunology exhibit the highest node density, suggesting their strong integration with Danshen studies. Four principal citation pathways were identified, demonstrating connections from Molecular Biology, Immunology, to Environmental Toxicology, Nutrition, Genetics, Health, Nursing, and Medicine. In addition, a distinct citation relationship was observed linking veterinary, animal, science studies, and molecular biology and genetics (Fig. 3C). Overall, the overlay visualization reveals the flow of knowledge across disciplines and highlights the central role of molecular biology and related fields in advancing Danshen research. Such citation patterns provide a deeper understanding of the interdisciplinary foundations of this field and point to future directions for scholarly development.

### 3.4. Author analysis

The analysis of authorship highlights the leading contributors and their academic impact in Danshen research. The team of Liang Zongsuo ranks first in publication output, with 70 articles and an *H*-index of 30, indicating that 30 of his publications have each been cited at least 30 times (Fig. 4A). His group has played a central role in systematically constructing the biosynthetic and metabolic framework of Danshen’s active compounds. In contrast, Zhou stands out in the co-citation analysis, with 791 co-citations (Fig. 4B). This high frequency reflects his work’s broad recognition and academic influence, underscoring his substantial contributions and scholarly authority. Together, these findings reveal not only the individual achievements of leading researchers but also the formation of core academic forces that drive knowledge dissemination and shape the intellectual structure of Danshen research.

### 3.5. Keyword and thematic analysis

**Figure 5.**
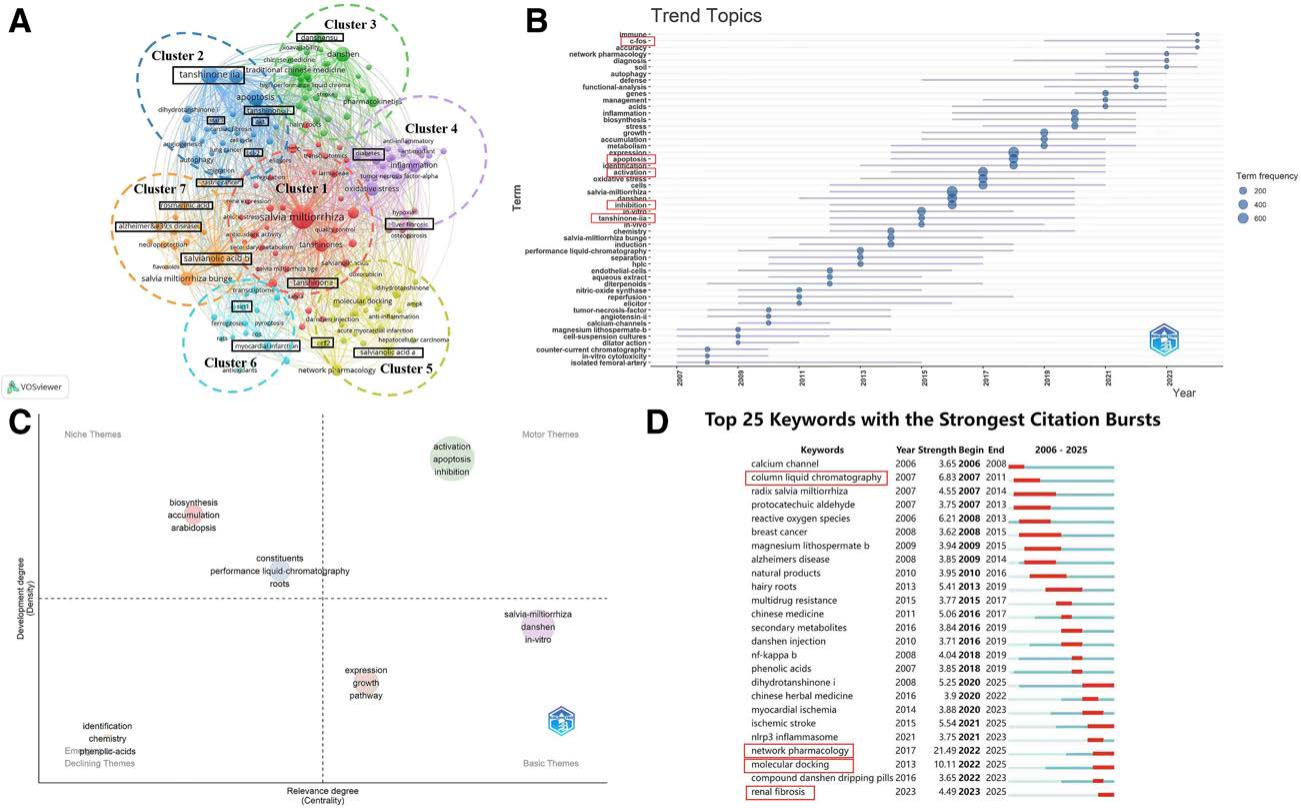
(A) Keyword co-occurrence network. (B) Temporal trend of keyword frequency, generated with Bibliometrix. (C) Thematic map showing research clusters, generated with Bibliometrix. (D) The top 25 keywords with the strongest citation bursts are visualized with CiteSpace.

Keyword and thematic analyses provide a comprehensive overview of the developmental trajectory and current hotspots in Danshen research. The field is gradually transitioning from traditional evaluations of medicinal efficacy toward mechanistic investigations at the molecular level.^[26]^ Based on the keyword co-occurrence network and temporal trend chart analyses, research hotspots are concentrated in 2 primary areas: the biosynthesis of active compounds and mechanistic studies related to clinical indications. Recent investigations increasingly emphasize molecular pathways, focusing on transcription factors such as Nrf2, Sirt1, and STAT3. Two dominant categories were identified for active compounds: tanshinones and phenolic acids. Tanshinone IIA, tanshinone, and tanshinone I were the most frequently studied among the latter. At the same time, salvianolic acid B, salvianolic acid A, and rosmarinic acid were the most common representatives of the latter, each appearing more than 45 times in the dataset (Fig. 5A, B). Notably, Liang Zongsuo’s group at Zhejiang Sci-Tech University has substantially contributed to biosynthetic research, particularly in elucidating these compounds’ metabolic synthesis.

In recent years, research on the clinical application of Danshen has become increasingly in-depth. Keyword clustering analysis indicates its therapeutic relevance across multiple disease categories, including cardiovascular conditions (e.g., myocardial infarction), neurological disorders (e.g., Alzheimer’s disease), liver diseases (such as liver fibrosis), metabolic conditions (notably diabetes), and several cancers (including gastric cancer). These areas are supported by more than 15 keyword occurrences, reflecting strong research interest and clinical potential. Of note, research on renal fibrosis has emerged only recently but is expanding rapidly, building on foundational work in diabetic nephropathy, suggesting a promising direction for future studies. Mechanistically, Danshen’s pharmacological actions are mediated by multitarget molecular networks involving signaling molecules such as C-FOS, Nrf2, and STAT3, and classical pathways including Akt, SIRT1, and BCL-2^[27,28]^ (Fig. 5A, B).

The thematic map of research clusters further illustrates the field’s structural development. Keywords such as “activation,” “apoptosis,” and “inhibition” appear in the upper-right quadrant, signifying their status as mature and stable research directions (Fig. 5C). Consistent with the thematic trend line chart, where these terms occur in over 200 publications, this highlights the central role of molecular interaction mechanisms in driving Danshen research (Fig. 5B). Keyword burst analysis provides additional insights. “Network pharmacology” exhibits the highest burst strength (21.49), followed by “molecular docking” (10.11) and “column liquid chromatography” (6.83; Fig. 5D). These results underscore the growing importance of advanced methodologies integrating computational and experimental approaches. Such tools facilitate the elucidation of compound accumulation patterns and pharmacological mechanisms at the molecular level while propelling TCM research toward greater precision and scientific rigor.

### 3.6. Citation and reference analysis

**Figure 6.**
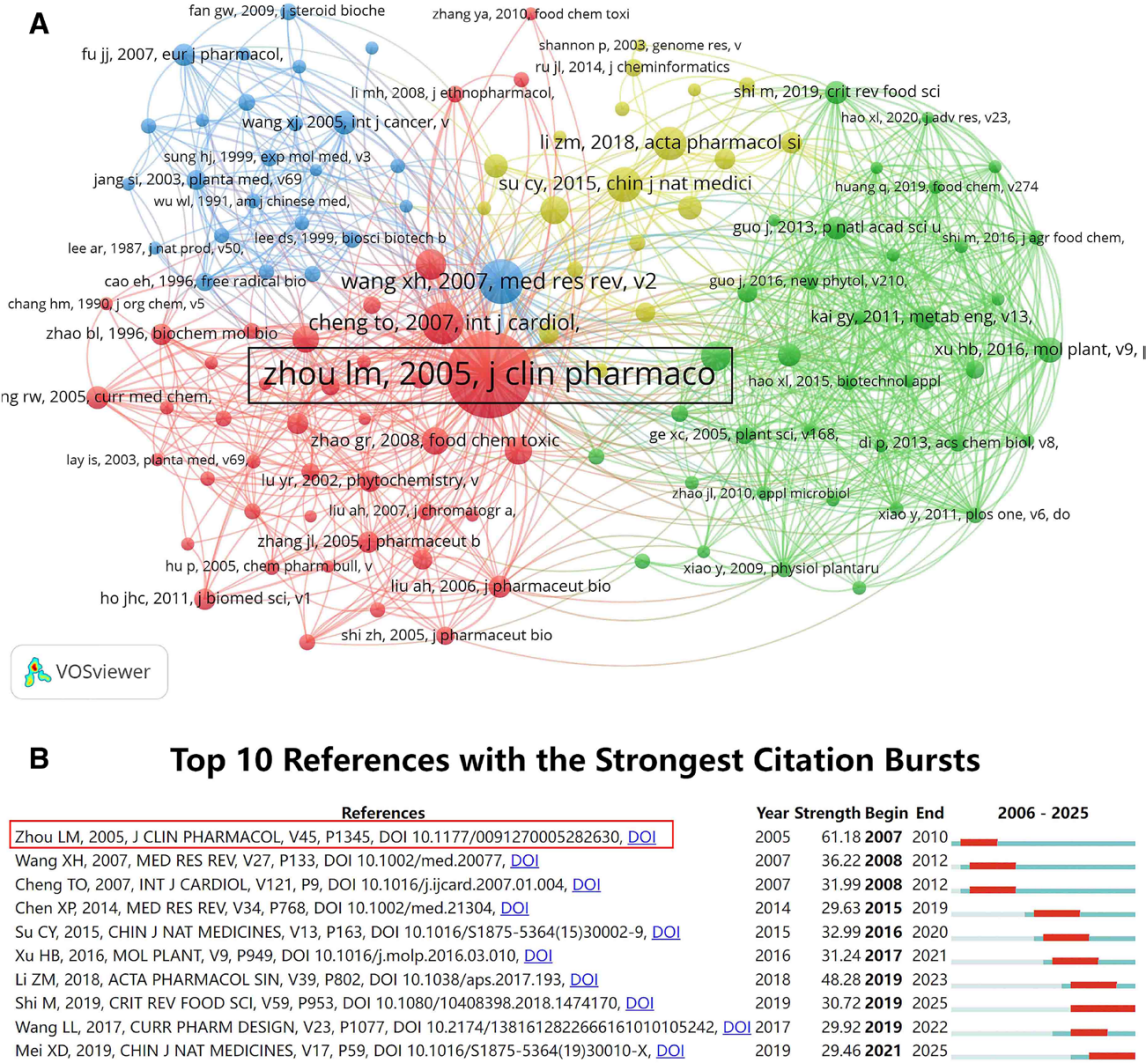
(A) Network visualization of co-cited references. (B) The top 10 references with the strongest citation bursts were visualized using CiteSpace software.

The co-citation network visualization (Fig. 6A) highlights the literature underpinning Danshen research. Among these, a landmark publication by Team of Zhou, published in the *Journal of Clinical Pharmacology* in 2005, emerged as the most influential, accumulating 696 citations at the time of analysis. Citation burst analysis further revealed that this study exhibited the most vigorous burst intensity, with a value of 61.18 (Fig. 6B). This pioneering work laid a solid theoretical foundation for subsequent investigations into multicomponent synergy, biosynthetic pathways, and clinical applications of Danshen, and has since been widely acknowledged as a milestone in the field.

A highly cited paper is one whose citation frequency ranks among the top 1% of documents in the same field. The data were collected in February 2025, and 13 highly cited publications in the Danshen field were identified, spanning biosynthesis, pharmacology, and materials science. Collectively, these studies have begun to establish an innovation chain that integrates gene mining, metabolic regulation, mechanistic exploration, and clinical validation, thereby providing a model framework for the modernization of TCM.

A substantial body of work has focused on the biosynthetic metabolism of tanshinones and phenolic acids. Researchers have elucidated key regulatory mechanisms using genomics, transcriptomics, and protein function analysis. For example, SmMYB98 has been identified as a molecular switch that simultaneously controls the synthesis of tanshinones and salvianolic acids.^[29]^ In tanshinone biosynthesis, enzymes such as CYP76AH3, CYP76AK1, and members of the CYP71D subfamily play essential roles.^[30,31]^ Next-generation sequencing and single-molecule real-time sequencing have further advanced mechanistic understanding.^[32]^ Among phenolic acids, SmERF115, a novel AP2/ERF transcription factor, was identified as a positive regulator of their production.^[33]^ In addition, methyl jasmonate (MeJA)-mediated signaling activates SmMYC2, which subsequently regulates SmbHLH60, coordinating the synthesis of phenolic acids and anthocyanins.^[34]^ These findings enrich the theoretical basis of tanshinone and phenolic acid biosynthesis while providing tangible molecular targets for metabolic engineering.

At the pharmacological level, progress has been made in elucidating the multitarget and synergistic mechanisms of Danshen-derived compounds. For instance, the quinone derivative miltirone selectively activates gasdermin E (GSDME) to induce GSDME-dependent pyroptosis, inhibiting liver cancer cell proliferation.^[35]^ Similarly, paeonol and danshensu combination (PDSS) was shown to mitigate isoproterenol (ISO)-induced myocardial infarction through activation of the NRF2/PI3K signaling pathway, leading to reduced oxidative stress and apoptosis.^[36]^ Together, these studies construct a “compound-target-pathway” regulatory framework, offering molecular evidence for the classical efficacy of Danshen in “promoting blood circulation and removing blood stasis” and facilitating the transition of TCM from empirical practice toward mechanism-based research.

Interdisciplinary integration with materials science has also yielded important advances. Electrospun nanofiber dressings incorporating Danshen extracts have been developed, promoting collagen deposition and angiogenesis, and reducing the healing time of diabetic wounds to 18 days.^[37]^ This represents a successful extension of Danshen-derived compounds beyond pharmaceuticals into biomedical device applications.

Additional highly cited works have systematically reviewed Danshen’s chemical constituents, quality control standards, and potential herb–drug interactions. Research on phenolic acids has further identified key biosynthetic enzymes and regulatory networks, enabling the construction of efficient synthetic biology platforms for their production.^[38]^ Moreover, phenolic acids and tanshinones’ biological activities and molecular mechanisms in treating blood stasis have been comprehensively summarized.^[39]^ Collectively, these integrative studies lay a solid scientific foundation for the advanced development of Danshen and offer a replicable methodological framework for the modernization of TCM.

## 4. Discussion

Our results demonstrated that publication output on Danshen has steadily increased over the past 2 decades (Fig. 1), reflecting the growing recognition of herbal medicine in global scientific discourse and the increasing integration of traditional remedies into modern biomedical research.^[9]^ The annual number of publications shows a relatively consistent upward trajectory, with noticeable acceleration in the last 5 years, suggesting a recent intensification of research efforts. Importantly, this trend is not merely quantitative; it represents a structural transformation in the research landscape. Studies on Danshen increasingly intersect with advanced scientific fields, including systems biology, molecular engineering, nanotechnology, and synthetic biology, signaling a shift from descriptive pharmacology toward mechanistic, translational, and applied investigations.^[19,34]^ This repositioning underscores Danshen’s emerging role as a bridge between centuries-old traditional knowledge and contemporary biomedical innovation, suggesting that the plant is now valued for its historical use and potential to inspire new therapeutic strategies.^[14]^

At the national level, our bibliometric analysis revealed that China, the United States, and South Korea are the primary contributors to Danshen research (Fig. 2). China’s leading position aligns with its long-standing clinical use of Danshen and strong governmental support for traditional medicine research, including dedicated funding programs and institutional initiatives.^[40]^ The United States’ significant contribution likely reflects growing interest in natural products for cardiovascular and oncology-related applications and investments in mechanistic and pharmacokinetic studies.^[41]^ South Korea’s active role underscores its emphasis on complementary and integrative medicine research.^[5]^ In contrast, relatively low output from Europe, Africa, and other regions highlights global disparities in research capacity, infrastructure, and funding. Such imbalances limit the diversity of scientific perspectives, potentially concentrating knowledge production within a narrow geographic scope. Expanding participation from underrepresented regions could promote a more balanced research ecosystem and facilitate the translation of Danshen into internationally relevant therapeutic applications.

Institutional and author-level analyses further highlighted these patterns. The Chinese Academy of Sciences and several leading universities ranked highest in productivity, with Liang Zongsuo emerging as the most prolific author regarding publication count and citation impact (Figs. 2 and 4). The top 5 institutions collectively contributed over 40% of the publications, emphasizing a concentrated but highly productive research base. Despite this domestic strength, collaboration network analysis revealed fragmented connections across institutions and authors, suggesting that much of the work remains siloed.^[42]^ Such weak ties may contribute to heterogeneity in experimental design, methodology, and reporting standards, limiting the comparability and reproducibility of findings. Lessons from other medicinal plants, such as ginseng and curcumin, demonstrate that international research consortia and multi-institutional collaborations are critical for advancing clinical development, harmonizing methodologies, and establishing standardized research protocols.^[43]^ International collaboration represents another crucial area for improvement. Although cross-border partnerships have increased modestly, coauthorship networks remain relatively weak and regionally clustered (Fig. 2).^[44]^ Limited collaboration restricts knowledge circulation, reinforces methodological fragmentation, and slows the establishment of standardized practices. Encouraging stronger international networks could facilitate cross-disciplinary expertise, increase methodological consistency, and improve the credibility of Danshen research on a global scale.^[45]^ Therefore, strengthening domestic and cross-border collaborations is essential to foster reproducibility, reduce methodological heterogeneity, and accelerate the translational potential of Danshen research.

Keyword co-occurrence and clustering analyses indicated that cardiovascular disease remains the dominant research theme (Fig. 5), consistent with Danshen’s historical and clinical use for circulatory disorders.^[28]^ The clusters corresponding to cardiovascular disease accounted for nearly half of all keywords, reflecting sustained interest in this field. However, emerging research hotspots, including fibrosis, oncology, and bioengineering, suggest significantly broadening the research spectrum. Danshen is increasingly investigated as a source of bioactive compounds across multiple disease contexts.^[46]^ Research on Danshen biosynthesis has evolved into a central focus of the field, advancing from early efforts to optimize natural drug extraction processes to strategies that enhance the accumulation of its bioactive constituents through environmental modulation, elicitor application, and regulation of key factors.^[47]^ The growing prevalence of pharmacokinetic studies, biosynthetic engineering, and compound optimization efforts further demonstrates a trend toward translational research, aiming to improve bioavailability, elucidate metabolic pathways, and establish scalable production systems.^[12]^ These developments align with broader trends in natural product research, where synthetic biology and metabolic engineering are leveraged to improve therapeutic accessibility, sustainability, and efficiency.^[48]^

Our analysis further identified 2 major issues: a disproportion between preclinical and clinical research, and a disconnect between experimental studies and large-scale cultivation. Laboratory investigations have advanced our understanding of molecular mechanisms, including anti-inflammatory, antioxidant, anti-fibrotic, and antiapoptotic pathways, but clinical trials remain relatively scarce. Existing trials often have small sample sizes, variable formulations, and heterogeneous endpoints, which complicates evidence synthesis and hinders the development of robust, real-world clinical recommendations.^[49]^ Specifically, <10% of the publications included controlled clinical studies, indicating a substantial gap between mechanistic research and translational application. This disconnect between mechanistic insights and clinical validation underscores the need for rigorously designed multicenter clinical trials using standardized preparations and harmonized outcome measures. Such trials should assess efficacy and safety and investigate pharmacokinetics, herb–drug interactions, patient-centered outcomes, and long-term effects to enhance clinical relevance and generalizability.^[50]^

Additionally, common challenges remain in the investigation of natural medicine molecule synthesis. Current research primarily emphasizes the isolated effects of individual environmental factors, elicitors, or regulators, whereas studies addressing multiple factors’ synergistic or interactive influences are comparatively limited.^[15]^ This limitation hampers a comprehensive understanding of the biosynthetic mechanisms of Danshen’s active constituents under natural growth conditions or complex industrial settings. While the superior clinical efficacy of perennial Danshen compared with short-term cultivated plants is recognized, most studies remain centered on short-term fluctuations in active component levels, with little systematic evaluation of plant growth status and medicinal safety under prolonged environmental stress or sustained regulatory induction.^[51]^ At the same time, biosynthesis research has remained mainly at the stage of laboratory validation, with limited progress in process development or practical approaches suited for large-scale cultivation and industrial application.^[52]^ The interplay of these factors has created a pronounced mismatch between research outcomes and the practical requirements for efficient production of medicinal constituents, resulting in a substantial barrier to translating findings into applications.

Burst analysis (Fig. 5) highlighted emerging trends in Danshen research, including omics technologies, network pharmacology, and nanomedicine. This indicates a shift toward systems-level approaches that clarify multicomponent interactions and improve drug delivery.^[53]^ Artificial intelligence and big-data analytics offer avenues to accelerate target discovery, optimize compound combinations, and predict therapeutic outcomes.^[54]^ Since 2020, research on pharmacokinetics and metabolic engineering has increased, focusing on translational applications. These methods may redefine how Danshen is studied and standardized, integrating traditional knowledge with advanced technologies to develop novel therapeutics and enhance clinical translation.

## 5. Limitations

This study has several limitations that should be acknowledged. First, only the Web of Science Core Collection was used as the data source, which may have excluded relevant publications indexed in other databases such as PubMed, Scopus, or China National Knowledge Infrastructure. Second, the analysis was limited to studies published up to February 2025; newer research was not captured and may alter the observed trends. Third, only original research articles were included, while reviews, conference abstracts, and patents were excluded, potentially narrowing the scope of analysis. Finally, the bibliometric tools applied in this study, including CiteSpace, VOSviewer, and Bibliometrix, rely on algorithm-based clustering and keyword co-occurrence, which can introduce biases depending on the search strategy and data cleaning process. Despite these constraints, using multiple software platforms and nearly 2 decades of data provides a reliable and informative overview of Danshen research.

## 6. Conclusion

Danshen research has grown substantially, with new applications emerging beyond cardiovascular disease. While preclinical studies are advanced, clinical validation, and international collaboration remain limited. Leveraging modern technologies and integrating traditional knowledge could accelerate translation and maximize Danshen’s therapeutic potential.

## Acknowledgments

Thanks to all study participants for their cooperation.

## Author contributions

**Conceptualization:** Huan-Huan Li, Fei-Fei Bu, Peng Wang.

**Formal analysis:** Huan-Huan Li, Chuan-Yao Zhang.

**Funding acquisition:** Peng Wang.

**Methodology:** Huan-Huan Li, Chuan-Yao Zhang.

**Software:** Chuan-Yao Zhang, Wen-Li Zhang.

**Supervision:** Chuan-Yao Zhang, Peng Wang.

**Visualization:** Fei-Fei Bu, Chuan-Yao Zhang.

**Writing – original draft:** Huan-Huan Li, Fei-Fei Bu.
